# Persistent LHPA Activation in German Individuals Raised in an Overprotective Parental Behavior

**DOI:** 10.1038/s41598-017-01718-z

**Published:** 2017-06-05

**Authors:** E. Ullmann, J. Licinio, A. Barthel, K. Petrowski, T. Stalder, S. R. Bornstein, C. Kirschbaum

**Affiliations:** 1 Department of Medicine, TU Dresden, Carl Gustav Carus, Dresden, Germany; 2 Department for Child and Adolescent Psychiatry, Psychotherapy, and Psychosomatics, University of Leipzig, Leipzig, Germany; 3 Mind and Brain Theme, South Australian Health and Medical Research Institute, Adelaide, Australia; 4 Department of Psychiatry, Flinders University, School of Medicine, Adelaide, Australia; 5 South Ural State University, Biomedical School, Chelyabinsk, Russian Federation; 6 Medicover, Bochum, Germany; 7 German Sport University, Cologne, Germany; 8 Department of Psychology, TU Dresden, Dresden, Germany; 9 Clinical Psychology, University of Siegen, Siegen, Germany; 10 Faculty of Life Sciences & Medicine, Rayne Institute, Endocrinology and Diabetes, Kings College London, London, UK

## Abstract

Parental upbringing may affect their offspring’s mental state across the entire lifespan. Overprotective parental child-rearing style may increase the disease burden in the offspring. Furthermore, this child-rearing style may also play a pathogenetic role by transmitting trauma- and stressor-related disorders (TSRD) across generations. Studies with animals have demonstrated that the mother’s immediate and expansive protection of the newborn decreases the limbic-hypothalamic-pituitary-adrenal (LHPA) axis activity in the offspring. However, few studies have investigated how stress impact humans raised in an overprotective manner. In a cross-sectional study with 40 healthy students recalling their overprotective upbringing, we show an increase in the dehydroepiandrostendione (DHEA) concentration and a reduction in the cortisol/DHEA-ratio in hair. Additionally, this child rearing style was associated with heightened indications of mental burden, depressiveness, and sense of coherence. Our results provide insight into the roots and consequences of psychological trauma across several generations. Further investigations focusing particularly on multigenerational transmission in extremely burdened families will augment our results.

## Introduction

War refugees and their risk of developing trauma- and stressor-related disorders (TSRD) require an improved understanding of these disorders^1^. Previous mental traumatization in TSRD patients exhibits an alteration in the limbic-hypothalamic-pituitary-adrenal (LHPA) axis activity^2^. The LHPA axis may experience a “load factor” during continual heightened arousal, whereby a negative feedback loop can maintain and enhance hypocortisolism and variability in dehydroepiandrosterone (DHEA) and cortisol/DHEA-ratio^3, 4^. A stronger anti-glucocorticoid effect of DHEA related to cortisol was used to explain lower cortisol/DHEA ratios in patients with TSRD^4^. A new biomarker using long-term hair steroid concentrations revealed an altered LHPA axis activity after a psychological traumatization without circa- and ultradiane rhythmics^5^. Intriguingly, these LHPA axis activity alterations and several TSRD symptoms may be transmitted over three generations, as found in families traumatized by the Holocaust^6–8^. Currently, the transmission mechanisms are being intensively examined and may provide an increased understanding for future development, treatment and prophylaxis of mental and psychological traumatization^9^.

Some studies describing these mechanisms are available in reference to the children of Holocaust-survivors who, in the aftermath of their own extreme physical and mental burdening, brought up their offspring with overprotective rearing measures^10^. The latter then led to a disturbed process of infantile individuation in the subsequent generation, where the child would frequently become the mother’s “mental container”, consequently developing into a disorganized mother-child attachment^11, 12^. Disorganized attachment is associated with pathological character traits following mental burdening in early childhood as well as atypical parenting^13, 14^. Perceived parental rearing behavior is linked to adult attachment patterns in intimate relationships^15^. The process of child separation-individuation and attachment is an integral part of early childhood development and it could be shown that frustrating attachment behavior is associated with depressiveness and TSRD symptoms as well as LHPA axis alterations up to old age^13, 16–18^.

Recently, a neural mechanism concerning parental behaviors has been described and the influence of parental behavior on their offspring’s physiology has been examined in animal experiments which found that extensive maternal care decreased the LHPA axis activity in their young^19, 20^. Yet there is a lack of evidence concerning the consequences in psychophysiology of intensive maternal care in humans. Interestingly, healthy Japanese students, who recalled overprotective parental rearing behavior, showed a hippocampal gray matter volume reduction associated with LHPA activity damping^21^.

Parental rearing behavior has a significant impact on the entire psychological development of the ‘thus raised’ and represents an important socialization factor in the formation of individual personality traits and attitudes^22^. Parental child-rearing behavior influences the entire lifespan of an individual far beyond the limits of childhood and adolescence^23^. Child-rearing practices, attitudes, and goals are classified within authoritative, permissive, and authoritarian child-rearing styles^24^. There is a high correlation in reference to the relevant child-rearing style dimensions in the literature when models with two or even three dimensions occur^15, 23^. These mostly factor-analytically gained dimensions are frequently characterized by conceptually opposing pairs and are understood, for the most part, as being independent of each other. By taking into consideration the various terminologies employed, the first pair is labeled affection (warmth, love) vs. rejection (hostility), and the second factor as control (overprotection) vs. autonomy (individuation)^15^. Some child-rearing styles are described as etiopathogenesis factors which arise from an abnormal condition or a disease^25, 26^. Subjects who recalled an overprotective rearing-style exhibited depression and anxiety and showed an increased prevalence of eating disorders^6, 27^. Moreover, in the third generation after the holocaust, individuals often reported a higher level of overprotecting parental rearing behavior with concomitant increased rates of psychosomatic symptoms^6^. Besides, subjects who reported higher levels of overprotective parental rearing were rather insecurely attached in romantic relationships^15^.

Consistent with the transgenerational pathogenic circumstances following emotional and psychological traumatization, the salutogenesis model has been receiving growing attention and consideration in regard to understanding stress-related disorders^28^. In this model, disease and health are not mutually exclusive categories but endpoints in the health-ease/dis-ease continuum, as a human being is not healthy or ill but rather more or less healthy or ill^29^. So, the focus lies on the sense of coherence (SOC), which is an essential coping resource for promoting stress resistance^28^. Especially in war-affected populations with an increased sense of insecurity, the symptoms of TSRD and depression are mediated by the SOC^30^. Moreover, a buffering effect of the SOC on the LHPA axis activity is indicated^31^. A lower SOC directly affects glucose tolerance and contributes to the onset of type 2 diabetes mellitus by inducing insulin resistance after LHPA axis activation^32^. Currently, no data exist concerning influences between the SOC and parental rearing styles. However, the SOC fully mediates the effects of parent and peer attachment on the depressive symptom level^33^. The function of parent and peer attachment bolstering competence supported the relationship between attachment and the SOC in three adolescent groups^34^. Due to the earlier described gap between anomalous parental behavior and disorganized attachment we predicted a lower SOC in respect to overprotective parental behavior.

The aim of this study was to examine the neurophysiological mechanisms, including depressiveness and sense of coherence, in the context of an overprotective parental style in the past in a normative sample. We predicted that the more the mentally burdened anxious mother uses her child as a containing object, the more she attaches her child with disorganized behavior through overprotective parenting, which impedes the child’s individuation process and causes a hyper-activation of the LHPA axis up to adulthood. Higher levels of depressiveness as well as lower abilities concerning the SOC indicate a higher mental vulnerability in thusly raised subjects.

Hereby, we examined new diagnostic options to substantiate the clinical routine psychiatric care for TSRD patients after global warfare, migration, and terrorism. In this context, we measured overprotective parental rearing styles as well as long-term steroid levels in hair as a valid biomarker. This biomarker approach is similar to the HbA1c-value in diabetes mellitus without circadian and ultradian variabilities, which is why we utilized this approach here^35, 36^. Correlating hair steroid concentrations with attachment-based overprotective parental rearing styles, depressiveness, and the SOC might validate these new markers in TSRD.

## Results

### Overprotective Parenting of Mentally Burdened Subjects

**Figure 1:**
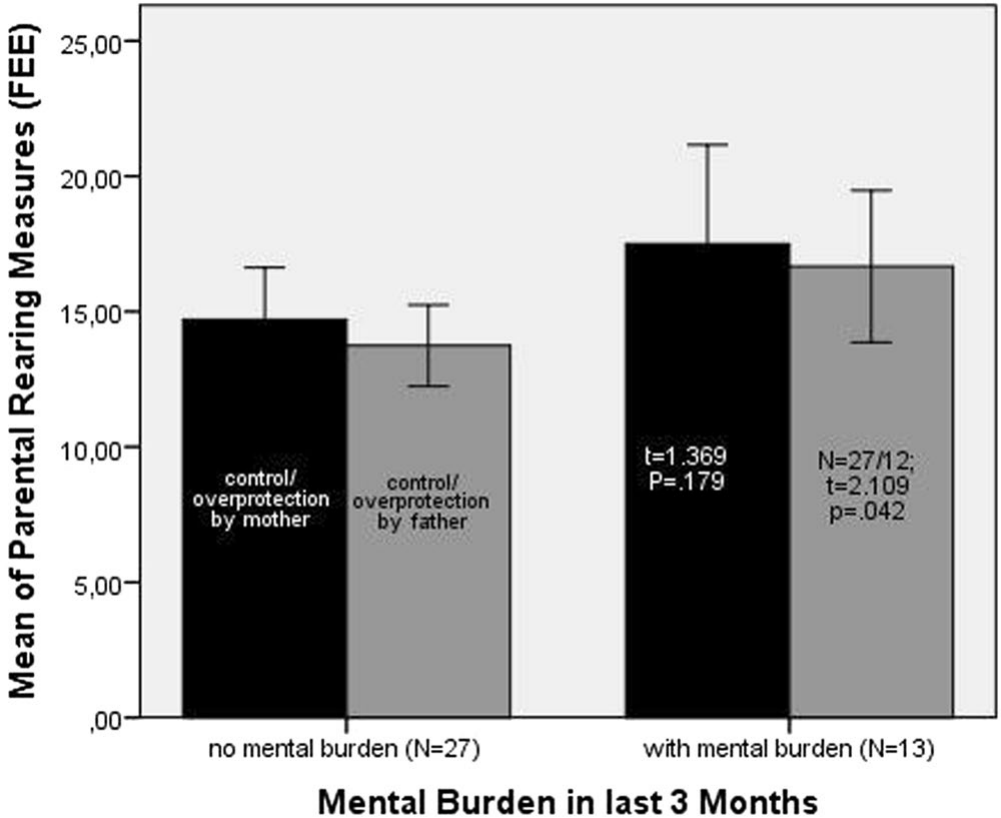
Parental child rearing styles of the FEE in subjects with and without mentally burdening events in the previous 3 months. Two-tailed test; error-bars 95%CI; One questionnaire of the paternal Recalled Parental Rearing Behavior Questionnaire (FEE) was missing. Paternal rearing measures of overprotecting (mean = 16.7 ± 4.4 SD, N = 12) compared to subjects without mentally burdening events (mean = 13.7 ± 3.8 SD, N = 27) in the previous three months. Maternal overprotecting in mentally burdened subjects (mean = 17.1 ± 5.7 SD, N = 13) compared to subjects without mentally burdening event (mean = 14.7 ± 4.8 SD, N = 27) in the previous three months.

Subjects who reported mentally burdening events in the previous three months more frequently recalled overprotective paternal rearing measures than subjects without mental burdening (two-tailed t-test, df = 37, t = 2.11, p = 0.042; Fig. 1). Also, subjects who reported mentally burdening events in the previous three months more frequently recalled (two-tailed t-test, df = 38, t = 1.37, p = 0.0179; Fig. 1) an overprotective mother. Moreover, the data of our whole group concerning recalled maternal and paternal overprotective rearing styles were almost identical (r = 0.857; p < 0.001) which is why we put them together and generated a new variable (“parental overprotectiveness”) for further calculations. The FEE of our whole group showed identical psychometric properties for maternal (mean = 15.5 ± 5.2 SD, N = 40) as well as paternal (mean = 14.6 ± 4.2 SD, N = 39) overprotective rearing measures compared to a representative sample of maternal overprotectiveness (mean = 15.4 ± 3.8 SD, N = 1471) and paternal overprotectiveness (mean = 14.9 ± 3.81 SD, N = 1423) in Germans^15^.

Concerning the physiological stress-markers, we found that subjects with mental burdening in the previous three months showed higher concentrations of cortisol (mean = 5.3 ± 0.7 pg/mg SE (N = 27)) in hair than subjects without mentally burdening events in the previous three months (mean = 3.4 ± 0.3 pg/mg SE (N = 27)) reaching the level of significance (two-tailed t-test, df = 16.9, t = 2.30, p = 0.03). Consistent with this, the subjects with the stated mental burden during the previous three months (mean = 0.14 ± 0.11 SD (N = 13)) showed a significantly higher (two-tailed t-test, df = 37, t = 2.96, p = 0.006) subjective stress level compared to the participants without a burden (mean = 0.03 ± 0.12 SD (N = 26)) as determined by the Perceived Stress Questionnaire (PSQ).

### Depressiveness and Sense of Coherence in Mentally Burdened Subjects

We found no significant differences in depressiveness (t = 0.78, p = 0.44) between subjects with (mean = 4.0 ± 3.4 SD, N = 13) and without (mean = 3.2 ± 2.3 SD, N = 27) mentally burdening events in the previous three months while the data of our whole group show identical psychometric properties compared to an age-controlled representative sample (mean = 2.9 ± 2.2 SD, N = 136) as determined by the HADS-D^37^.

The SOC-9L in our whole group (mean = 45.3 ± 6.5 SD, N = 40) showed identical psychometric properties compared to a representative sample (mean = 47.5 ± 8.9 SD, N = 2005) whereas subjects with a mental burden showed a lower ability to the sense of coherence (mean = 42.5 ± 7.2 SD, N = 13) than subjects without memories of mentally burdening events in the previous three months (mean = 46.6 ± 5.8, N = 27) without reaching the level of significance (t = 1.8; p = 0.09)^38^.

Concerning the hair- and stress-related control variables, there were no differences between subjects with and without mentally burdening events in the previous three months, including age, gender, frequency of hair washing, alcohol consume/week, BMI, and anxiety (Table 1).

**Table 1:** Sociodemografic data of stress- and hair- related characteristics in subjects with and without mentally burdening events in the previous 3 months.

	Whole group (N = 40)		Subjects without mental burdening (N = 27)	Subjects with mental burdening (N = 13)	Sig.
	mean ± SD	range	mean ± SD	mean ± SD	p
Age	24.1 ± 4.4	18–35	24.3 ± 3.7	23.6 ± 5.8	0.70
Sex (frequencies)	^♀^=22	^♂^=18	^♀^=16 ^♂^=11	^♀^=6 ^♂^=7	*x* ^2^ = 0.44
Frequency hairwashing (N = 32; 23/9)	4.8 ± 2.0	1–7	4.7 ± 1.9	4.8 ± 2.3	0.96
Alcohol consume/week	3.1 ± 0.8	1–5	3.2 ± 0.9	3.0 ± 0.7	0.51
Body Mass Index (N = 10; 3/7)	22.5 ± 4.8	18–33	19.6 ± 1.4	23.8 ± 5.2	0.22
Anxiety (HADS-subscale)	6.3 ± 3.5	1–18	5.7 ± 2.4	7.6 ± 4.9	0.10

### Overprotective Parenting, LHPA Axis Activity and Depressiveness and SOC

**Figure 2:**
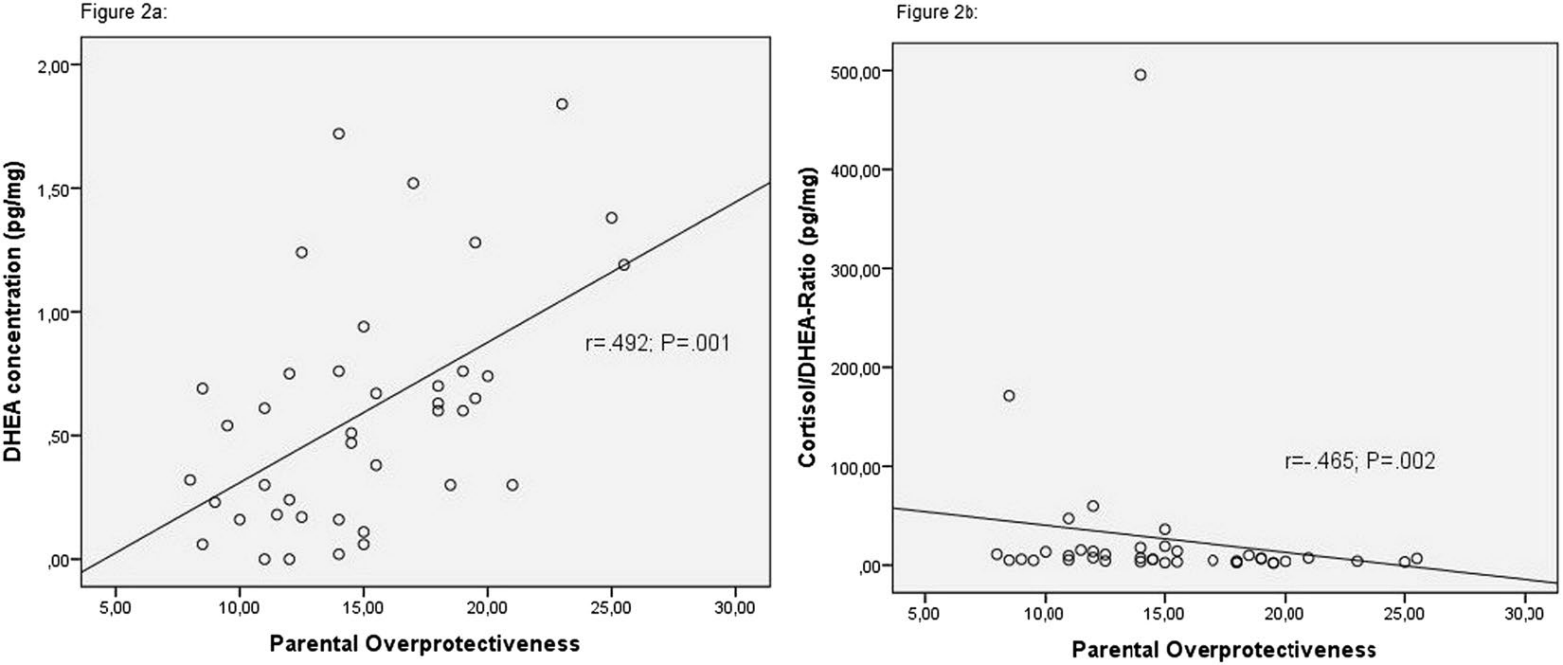
Spearman’s correlations between parental child rearing practices and the long-term LHPA axis activity. Figure 2a shows Spearman’s correlations between dehydroepiandrostendione (DHEA) concentrations and parental overprotecting. Figure 2b shows Spearman’s correlations between cortisol/DHEA-ratio and parental overprotecting. Cortisol/DHEA-ratio was calculated by using the concentrations of cortisol and DHEA in 3 cm hair segments. The level of “parental overprotecting” was generated by using the mean-values of maternal and paternal parental overprotecting of the Recalled Parental Rearing Behavior Questionnaire (FEE).

**Figure 3:**
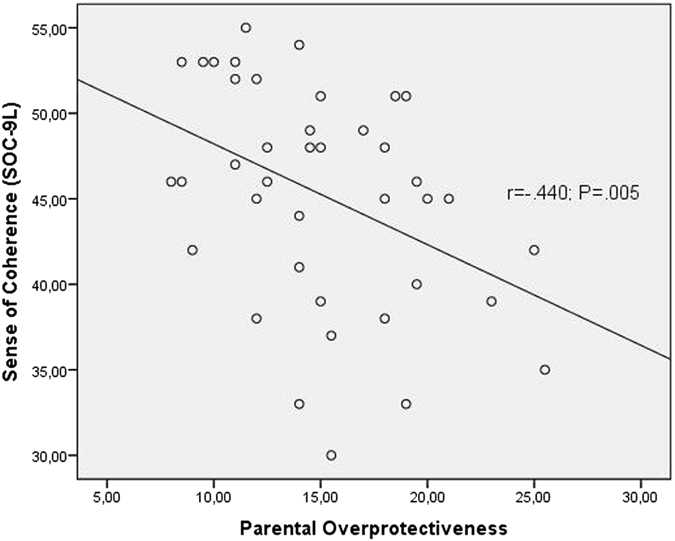
Spearman’s correlations between sense of coherence and parental overprotecting. The level of “parental overprotecting” was generated by using the mean-values of maternal and paternal parental overprotecting of the Recalled Parental Rearing Behavior Questionnaire (FEE). The level of sense of coherence was captured by using the Sense of Coherence Scale (SOC) in its abbreviated form of nine items (SOC-9L).

**Figure 4:**
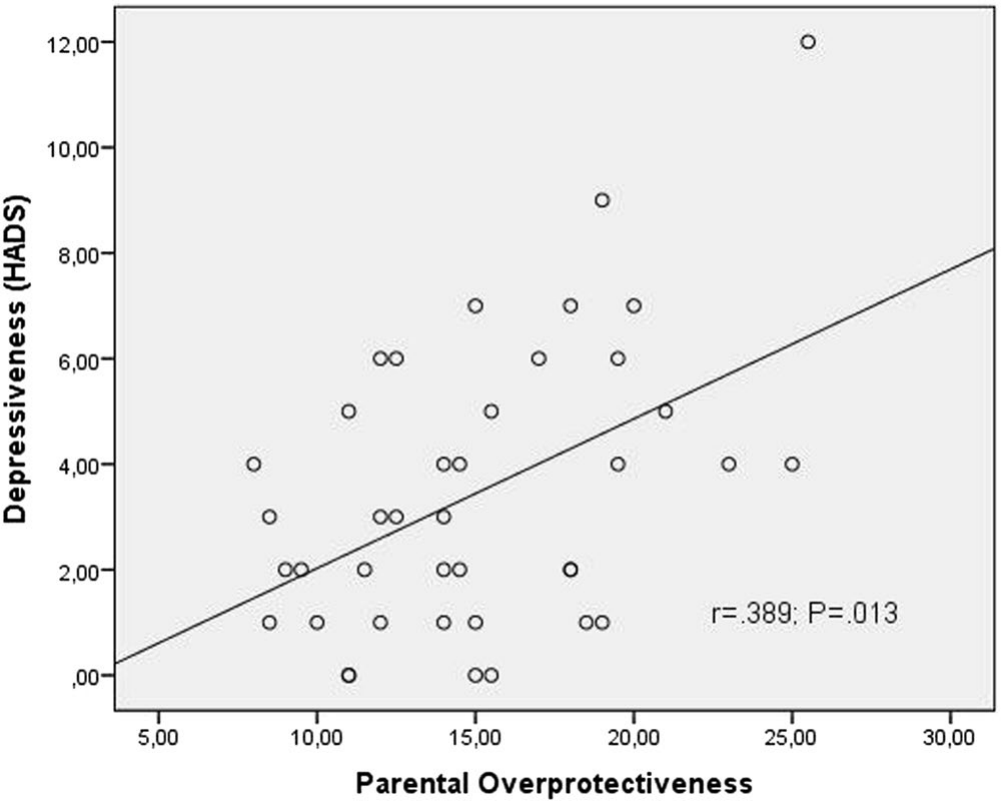
Spearman’s correlations between depressiveness and parental overprotecting. The level of “parental overprotecting” was generated by using the mean-values of maternal and paternal parental overprotecting of the Recalled Parental Rearing Behavior Questionnaire (FEE). Depressiveness was captured by using the hospital anxiety and depression scale (HADS).

Higher levels of our newly generated variable “parental overprotectiveness” were associated with higher levels of long-term DHEA-secretion (r = 0.492; p = 0.001, Fig. 2a) and lower levels of long-term cortisol/DHEA-ratio (r = −0.465; p = 0.002, Fig. 2b) but not with subjective stress perception measured by the PSQ (r = 0.128, n.s.). Higher levels of “parental overprotectiveness” were also associated with higher levels of depressiveness (r = 0.389; p = 0.01; Fig. 3) but not with anxiety (r = −0.026; n.s.). A lower ability for the sense of coherence accompanied more reports of overprotective parental rearing styles (r = −0.440, p = 0.005; Fig. 4).

## Discussion

To our knowledge, the present study examined the relationship between parental rearing and mental vulnerability with long-term LHPA axis alterations and personal salutogenic abilities in humans for the first time. In a sample of 40 German students, we showed that subjects with mentally burdening events in the previous three months reported higher levels of overprotective parental rearing behavior and perceived stress, including LHPA axis activation. The subjective experience of overprotective parental rearing (nurture) accompanied an physiological activation of the LHPA axis (nature) as well as higher levels of depressiveness and reduced personal moderating capabilities (SOC).

The conditions of mental development in the first year of life and the clinical outcomes of inadequate bonding in early childhood were sufficiently substantiated in reference to the attachment paradigm, and a gap was shown between unresolved loss, anomalous parental behavior, and disorganized infant attachment^13, 14^. Disorganized bonding is associated with LHPA axis activation, increased depressiveness as well as a reduced SOC^13, 18, 33^. Moreover, alterations in glucocorticoid receptor gene methylation are associated with mental burdening in early childhood, and, in turn, fearful attachment is associated with TSRD symptoms^16, 39^.

Moderate meta-analytical effect sizes indicate associations between anomalous parenting and disorganized parent-infant dyads, but there is a lack of evidence concerning the overprotective rearing style in persistent mental- and stress-loaded parent-infant relations^14^. In a representative German sample, recalled overprotective child rearing was associated with insecure attachment, and subjects who recalled an overprotective rearing-style exhibited depression and anxiety as well as showing an increased prevalence of eating disorders and psychosomatic symptoms^6, 15, 27^. Our data provide an insight into the psychophysiological impacts of overprotective child rearing as associated with depressiveness, mental burdening, and the sense of coherence as well as an LHPA axis activation.

Moreover, our data indicate the consequences of frustrated infantile individuation in early child development. It is often implied that a high degree of parental protection promotes the obstruction of infantile individuation in the attachment process, which in itself creates a pathogenetic component^10–12, 17^. While the attachment theory focuses primarily on secure mother-child relations from birth, a similar perspective with an increased accentuation of the individuation process has been discussed to a lesser degree^17^. In the matrix of mother-infant dyads, the child must actively perform the differentiation of body schemas to establish distance and demarcation as an individual. The child’s fresh and pliable adaptive capacity for gaining satisfaction is always greater than that of the mother whose personality with its patterns of character and defense is firmly and often rigidly set in spite of adapting herself she may make to the child to express sensitivity and empathy^40^. However, an inhibited individuation process through parental overprotection is associated with the maternal need to use her child as a “mental container”. Since the mother must keep her containing object, she often impedes the individuation process^10, 12^. Usually, containing describes the ability of the mother to deal with her child’s thoughts and feelings without an emotional or anxious reaction^41^. But in anxiety-related mentally burdened mothers, this process may reverse; the mother then successfully uses her child to contain her anxieties, which the child is unable to prevent^12^.

Interestingly, chronic LHPA activation patterns in our subjects contrast with results in Japanese students who recalled increased overprotective parental rearing^21^. The Japanese students showed a significantly lower level of LHPA axis activation and reduced hippocampal gray matter volume whereas the German students in our study showed significantly higher levels of long-term LHPA axis activation. We used concentrations of long-term hair steroids without circa- and ultradiane rhythmics as a biomarker of traumatization in healthy individuals and TSRD patients^5^. Additionally, we used the cortisol/DHEA ratio from hair concentrations because a stronger anti-glucocorticoid effect of DHEA related to cortisol was used to explain lower cortisol/DHEA ratios in patients with TSRD^4^. Hence, we interpreted the inverse reaction patterns of the LHPA axis as a load factor following recurrent hyper-activation in the Japanese students as an adaptation of the LHPA axis to anxiety-related environmental conditions resulting in demethylation in gene expression^39, 42^. However, cross-cultural differences reflect the preference for “internal” and “external” locus of parental control, which needs to be considered^43^. Cultures highly valuing individual autonomy and self-actualization might elicit a reactive pattern of the LHPA axis activity to strong parental devotion, leading to an increased mental burden. Our results indicate this since higher levels of parental overprotection are associated with higher levels of depressiveness. Further evidence is needed concerning the etiopathogenesis of overprotective child rearing practices based on sensitive tests like an excretion of cortisol in the urine over 24 h or saliva cortisol concentration profiles^44–46^. And, further studies ought to determine psychobiological stress responses by using the Trier Social Stress Test (TSST)^47^ in a longitudinal, cross-cultural, attachment-based design with a larger sample.

Additionally, we were able to show a connection between parental rearing and the health-stabilizing SOC. Thus, individuals who recalled an increased overprotective parental rearing behavior showed less capability in their sense of coherence in regard to the health-ease/dis-ease continuum as well as the attachment paradigm. This decreased sense of coherence was accompanied by increased depressiveness, which is well supported by the existing literature and was recently substantiated by Chinese American students - the sense of coherence fully mediated the effects of parent and peer attachment on the depressive symptom level^33^. Especially in subjects with an exposure to traumatic events, the SOC may mediate symptoms of TSRD and depression^30^. The influences of the sense of coherence on the LHPA axis activity permit this presumption^31, 32^. Qualitatively designed studies (for example a home visit sequence analysis) should be used in perspective to find other key predictors of transmission mechanism in early childhood development of anxious-related mother-infant dyads whereas structured observations confront the mother and child with arousal stimuli^24^.

## Conclusions

We have established an enduring connection between parental rearing and mental burden which alters depressiveness and SOC as well as long-term LHPA axis activity. We have also provided further evidence that hair steroid measurements as well as overprotective parental rearing are reliable markers for clinical applications for the diagnosis of TSRD since we found a significant correlation between our quantitative self-report questionnaire responses and hair steroid levels. Both have never been included in clinical routine until now, which should happen in perspective, especially to capture the level of chronification after recurrent mental burdening over several generations.

## Limitations

One limitation of our study was utilizing a retrospective analysis of parental rearing. The FEE reliably surveys subjective representations of parental rearing behavior. The methodology used in our study does not assess actually observed parental rearing. Rather, it assesses perceived parental rearing behavior from childhood of recollections in adulthood. Measurements of the attachment style would have substantiated our results more strongly.

## Methods

Our cross-sectional study was approved by the IRB of the medical faculty Carl Gustav Carus at the Technical University (TU) Dresden. The study was approved in accordance with the Guidelines approved by the IRB in January 2015. Each participant provided signed informed consent according to the description of the study. Due to the conditions of the standardized questionnaires used, we included subjects between 18–35 years of age. Also, we avoided psychosocial transgenerational transmission influences following World War II as well as acculturation effects by including subjects without a prior immigration background as far back as two generations. We excluded subjects whose parents were born before May 8, 1945 and grandparents born after this date since those factors might also be stress-related. Subjects with known Cushing’s or Addison’s disease as well as hypo-/hyper-thyroidism or other known endocrine disorders were excluded for the same reason by finding out before starting the investigation. German subjects usually know about their hormonal diseases as there is a routine metabolic screening 36–72 h after birth which, for example, includes the activity of the 21-hydroxylase by measuring the concentration of 17-hydroxyprogeterone which physiologically accumulates in congenital adrenal hyperplasia. Questionnaires were filled out by the participants, and hair samples were taken. After this procedure, each participant received a compensation of 10 euros to cover expenses.

The subjects were characterized (Table 1) with regard to the following criteria: socio-demographic, stress-related characteristics (body mass index, alcohol consume, physical activity, depressiveness, anxiety, sense of coherence, resilience, and mentally burdening events during the previous three months) and hair-related characteristics (frequency of hair washing), as well as memories of parental child rearing practices (Table 1).

Hair strands (~3 mm diameter) were taken from the scalp near a posterior vertex region. Steroid hormone concentrations were determined in the proximal 3 cm - long hair segment which, based on an approximate hair growth rate of 1 cm per month^48^, reflects the integrated hormone secretion over the three-month-period prior to hair sampling. The concentrations of cortisol and DHEA were determined by liquid chromatography tandem mass spectrometry (LC-MS/MS) following our published protocol with 7.5 mg whole, non-pulverized hair used for the current analyses^36^.

The Recalled Parental Rearing Behavior Questionnaire (Fragebogen zum erinnerten Elterlichen Erziehungsverhalten, FEE) is the shortened German version of the Swedish questionnaire “Egna Minnen Beträffande Uppfostran” (My memories of upbringing, EMBU)^49, 50^. The EMBU was originally created to evaluate adult memories concerning their upbringing and is comprised of factor-analytically derived dimensions of (a) rejection and punishment, (b) emotional warmth, (c) control and overprotection. The EMBU as well as the FEE are standardized questionnaires for the assessment of three highly interrelated dimensions of recalled parental rearing behavior for each parent using 24-items with four scaled answers. Paternal/maternal control and overprotection assesses the parental behavior the child perceived as overly thoughtful, blaming, interfering, and constricting, which reflects a distinct orientation toward effort, performance, and high expectations by the respective parent. Questions, for example, are worded as follows: “Do you think, your mother/father was an anxious person, and therefore you were not allowed to do as many things as other children” or “Do you think, your mother/father felt exaggeratedly anxious that something bad might happen to you?”. There are high correlations between the perceived parental rearing behavior and insecure adult attachment patterns in intimate relationships, especially concerning controlling and overprotective parental practices^15^. The German version, both the items and the three scales, showed satisfactory to good psychometric properties (Cronbach’s Alpha > 0.72)^15, 51^.

Depressiveness and anxiety were recorded using the Hospital Anxiety and Depression Scale (HADS)^52^. In this standardized self-administered 4-point Likert scale, anxiety and depression are specified in two subscales generated from 7 items each, and an anxiety and a depression subscale is established. Questions are worded as follows: “I feel tense or irritable” or “I look happily forward to the future”. Higher scores indicate higher levels of anxiety or depressiveness. Current psychometric data validate the quality of this scale on the national and international level (Cronbach’s Alpha > 0.80)^37, 53–55^.

The participants’ SOC was investigated by the standardized SOC-scale in its abbreviated form of nine items (SOC-9L)^29, 38^. Within the framework of Antonovsky’s salutogenetic model, the SOC has an important role as a personal trait for the health-promotive outlook for dealing with stressors, including the activation of generalized resistance resources^56^. The SOC is described as an outlasting element across the life span and in a socio-cultural context. Using the SOC-scale, a general life orientation in the dimensions “comprehensibility”, “manageability”, and “meaningfulness” are recorded at different degrees of variation. Questions are worded as follows: “How often are your feelings and thoughts confused?” or “Do you expect for the future that your life will be without any sense and purpose – full of sense and purpose” and must be answered by using a seven-point Likert scale. Both, the SOC-scale as well as its abbreviated form (SOC-9L), are valid scientific measurements (Cronbach’s Alpha > 0.87)^29, 38^.

For example, the individual non-standardized questions are worded in the following manner. Did you have to carry an extraordinary mentally burdening event in the previous three months (yes/no)?- If yes, which? How actively have you been engaged in sports (1 = hardly at all to 10 = very active)? How much physical activity did you have on the whole (1 = very little to 10 = very intensive)? Also the data regarding age, sex, alcohol consume/week, hair-washing and BMI were captured by using non-standardized questions.

To control the stress perception widely, we used the 30-items Perceived Stress Questionnaire (PSQ)^57^. The PSQ allows for the quantitation of subjective perception, evaluation, and further processing of stressors by using scaled questions such as: “you find yourself in situations of conflict” or “your problems seem to be piling up”. Dominance and external stressor experienced during the previous four weeks can also be quantified by this test. The PSQ meets the highest national and international quality standards (Cronbach’s Alpha > 0.85)^57^.

After data entry in IBM SPSS Statistics 23, we adjusted for statistical outliers and determined standard deviations (Kolmogorov Smirnov) with normal distribution concerning all the data used in this study. To analyze the data concerning the LHPA axis activity, SOC and depressiveness, we generated a scale named “Parental Overprotectiveness” using the subscales of maternal and paternal rearing practices. Finally, we calculated the two-tailed test and spearmen’s-rho correlations.
